# Motor-language links in children with Down syndrome: a scoping review to revisit the literature with a developmental cascades lens

**DOI:** 10.3389/fpsyg.2023.1275325

**Published:** 2023-10-02

**Authors:** Atefeh Karimi, Eliza L. Nelson

**Affiliations:** Department of Psychology, Florida International University, Miami, FL, United States

**Keywords:** Down syndrome, motor, language, developmental cascades, scoping review

## Abstract

**Background:**

Children with Down syndrome (DS) typically have motor and language needs. Improving function is a shared goal for the rehabilitation therapy team, however physical therapy, occupational therapy, and speech-language pathology professionals treat patients differently. This difference in care may mask *developmental cascades* whereby changes in one domain (e.g., motor) can have seemingly unexpected effects on another domain (e.g., language).

**Objective:**

This scoping review identified papers where motor and language data have been reported together in children with DS and reinterpreted findings from a developmental cascades lens.

**Design:**

Online databases were used to identify 413 papers published before October 2021 from which 33 papers were retained that reported both motor (gross and/or fine) and language (expressive and/or receptive) data in individuals with DS with a chronological age of 0–18 years.

**Results:**

The majority of papers (79%) that reported motor and language data in children with DS did not examine their link, while 12% analyzed motor-language links, but using a cross-sectional or retrospective design. Only three papers (9%) utilized a longitudinal design to examine predictive links.

**Conclusion:**

Motor functioning and language functioning have often been reported together, but not analyzed together, in studies of children with DS. The few studies that did analyze motor-language links largely replicated findings from other developmental populations where motor gains were positively linked to language gains. Analyzing links between domains when such data is available is needed to fully characterize developmental cascades in DS and may have broad clinical implications.

## Introduction

Down syndrome (DS) has received considerable research attention relative to other genetic disorders with prior research focusing on strengths and needs in individual developmental domains (Fidler, 2005). Two of these well-studied domains in DS are motor functioning (Palisano et al., 2001; Needham et al., 2021) and language functioning (Abbeduto et al., 2007; Roberts et al., 2007; Martin et al., 2009). Children with DS who have motor and language needs are treated by a rehabilitation therapy team, which can include physical therapy, occupational therapy, and speech-language pathology professionals among other stakeholders. While this team has a shared goal of improving function for the child with DS, different team members address the child’s needs in each domain. Similarly, intervention research has focused on within-domain gains, such that motor interventions have tracked motor outcomes and language interventions have tracked language outcomes (for meta-analyses, see Smith et al., 2020; Ku and Sung, 2022). Taken together, there has been a trend to focus on discrete developmental domains in characterizing and treating children with DS.

However, there is a growing interest in cross-domain interactions in developmental research. Changes in one domain can have seemingly unexpected effects on another domain—a theoretical concept known as *developmental cascades* (Masten and Cicchetti, 2010; Oakes, 2023). An area of burgeoning research in child studies is motor-language cascades, whereby a gain in motor skill has downstream effects on language development (Iverson, 2010; Gonzalez et al., 2019; Iverson, 2021, 2022). Skills like the transition to independent sitting, independent walking, and complex object manipulation all dramatically change how the child acts in their environment, but also reciprocally, how caregivers respond to and engage with the child, including the language input they provide.

Evidence for developmental cascades is not limited to studies of typically developing children. The achievement of motor milestones has similarly been tied to language skills in neurodevelopmental disorders like autism spectrum disorder (ASD), developmental coordination disorder (DCD) and developmental language disorder (DLD; formerly Specific Language Impairment or SLI) (for reviews, see Leonard and Hill, 2014; Bradshaw et al., 2022). Further, a recent scoping review relative to the current study by Hwang and Lee (2022) examined how motor and language abilities are linked developmentally in ASD. These authors identified 11 papers on this topic over the past 20 years, finding a positive link between early motor skills and expressive and receptive language in ASD, but disagreement in assessment methodology. We are unaware of any similar review in DS on motor-language cascades, but such an approach is needed given both syndrome-specific features and within-syndrome variability in motor skills and language outcomes in this population. As a first step, we conducted a scoping review to map the potential evidence for motor-language cascades in DS.

## Methods

The protocol for this review was drafted using the Preferred Reporting Items for Systematic Reviews and Meta-analyses extension for scoping reviews (PRISMA-ScR) (Tricco et al., 2018; McGowan et al., 2020) and published on OSF on November 24, 2021.^1^ The review was conducted in accordance with JBI methodology for scoping reviews (Aromataris and Munn, 2020), and data extraction was based on the Arksey and O'Malley (2005) framework.

### Inclusion and exclusion criteria

To be included in the review, papers needed to measure both motor functioning (gross and/or fine or a combined score) and language functioning (receptive and/or expressive vocabulary) in children with DS with a chronological age of 0–18 years, regardless of whether the authors examined motor-language links. Papers were excluded if they only reported data for one domain (i.e., motor or language), if they were not peer-reviewed, if they were not written in English, if data from children with DS were pooled with data from another developmental population for analyses, or if participants were outside the target age range. There were no restrictions on date of publication or type of study design.

### Search strategy

**Figure 1:**
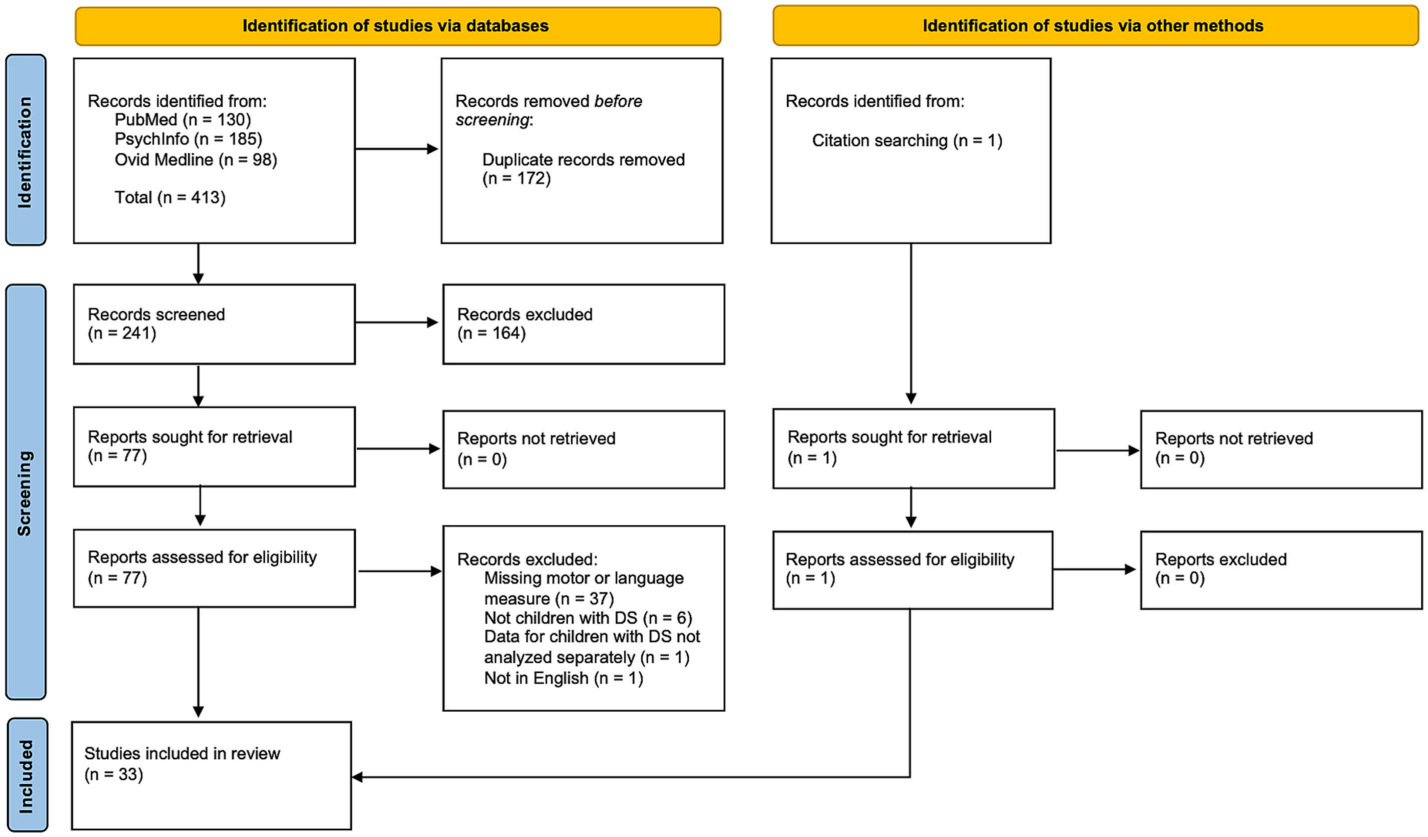
Study selection process.

Records were identified from searches conducted in October 2021 in three databases (PubMed, PsychInfo, and Ovid Medline) as well as through citation searching. Search terms included “Down syndrome” AND “motor” AND “language.” MeSH terms were included to maximize the sensitivity and specificity of the search in PubMed. Full search strings can be found in Supplementary Table S1. Search results were uploaded to Abstrackr, which is an online tool for organizing results in a review (Wallace et al., 2012). Both authors (AK and ELN) independently screened the titles, abstracts, and keywords of identified papers using the eligibility criteria with Abstrackr. The full text was then obtained for titles marked “yes,” “maybe,” or where reviewers disagreed on classification. Disagreements between reviewers were resolved through discussion. Figure 1 shows the full study selection process.

### Data charting and synthesis

Data were extracted from eligible studies using a chart developed by both authors through an iterative process, including the number and chronological age of children with DS, study location, and study design. Data extraction was performed by the first author (AK) with consultation from the second author (ELN). Data were charted based on whether the author(s) examined link(s) between motor and language data. For studies that reported motor and language data but did not examine motor-language link(s), data extraction additionally included the original author(s) study aims and the availability of motor and language data using yes/no coding for gross motor, fine motor, expressive language, and receptive language measures. For studies that did report a motor-language analysis, data extraction was more detailed and included the specific motor and language variables used in analyses with motor-language links coded as positive, negative, or none. Finally, a narrative synthesis was used to reinterpret the reported motor-language links from a developmental cascades lens.

## Results

A total of 413 records were identified from database searching. After duplicates were removed, 241 records were screened, of which 164 were excluded based on inclusion/exclusion criteria. The remaining 77 reports were retrieved and assessed for eligibility. An additional 45 papers were excluded during full-text screening because they were missing a motor or language measure (*n* = 37), data were not from children with DS (*n* = 6), data for children with DS were not analyzed separately (*n =* 1), or the paper was not in English (*n* = 1). One additional paper was identified from citation searching. The final number of studies included in the scoping review was 33 (see Figure 1).

### Most studies reporting motor and language data did not investigate the link between domains

The largest group of studies reviewed was author(s) who reported motor and language data in children with DS but did not investigate the link between these two developmental domains (79%; 26 of 33 papers). Details of these studies, published 1969–2020, are in Supplementary Table S1. The number of children with DS per study varied widely from 1 to 612, and their ages ranged from 0–18 years. Data were from 11 countries, but studies were disproportionally from the United States (12 of 26 papers). There were three studies each from the United Kingdom and Spain. All other countries contributed one study (in alphabetical order: Brazil, Canada, Chile, Japan, New Zealand, Norway, Taiwan, and Thailand). The most common study design was cross-sectional (*n* = 9) followed by longitudinal (*n* = 6). The remaining designs were matched pairs (*n* = 3), experimental (*n* = 2), randomized control trial (*n* = 2), retrospective (*n* = 2), prospective (*n* = 1), and case study (*n* = 1). Among these studies, 85% reported gross motor data, 92% reported fine motor data, 96% reported expressive language data, and 92% reported receptive language data. The studies varied in the assessments used to measure motor and language functioning given the wide range in ages within this grouping, as well as the scope of the original author(s) work. Most studies in this grouping could be categorized into one of three themes: (1) studies that focused on creating developmental charts or milestones, which may have made direct comparisons to typically developing children; (2) studies that examined treatment effectiveness (e.g., massage therapy) on general development; or (3) studies that compared groups of children with DS based on the presence or absence of a comorbid condition (e.g., congenital heart disease).

### Most studies reported positive findings when motor-language links were analyzed

Authors who reported motor and language data in children with DS and investigated motor-language links comprised a smaller proportion of studies reviewed (21%; 7 of 33 papers). Details of these studies are in Table 1. More than half of the papers were published in the past 5 years, suggesting an emerging interest in this topic. Most data were from the United States (*n* = 3), followed by Japan (*n* = 2), Canada (*n* = 1) and Italy (*n* = 1). Studies that were cross-sectional or retrospective in their design or analyses reported on concurrent motor-language links (*n* = 4), whereas studies that included a longitudinal component additionally reported on predictive motor-language links (*n* = 3). Designs were further considered in the context of how motor functioning was measured using (a) one of three global assessments (BSID, KSPD, or MSEL; see Table 1 for key) or (b) one of four individual motor variables (muscle tone, finger repetition, sitting onset, or walking onset). Language functioning was measured from one of six global assessments (KSPD, PPVT-R, REEL, RSLD, VABS-II, or WPPSI-III; see Table 1 for key). One study additionally used babbling onset as a variable in analyses.

**Table 1:** Papers that reported motor and language data in children with Down syndrome and analyzed motor-language links.

Source	*N*	CA	Setting: design	Motor variable	Language variable	Motor-language link
Reed et al. (1980)	89	0–36 months	US: longitudinal	FM/GM BSID (6, 12, 18, 24, 30, 36 months)	EL REEL (36 months)	+
				FM/GM BSID (6, 12, 18, 24, 30, 36 months)	RL REEL (36 months)	+
				GM Muscle Tone (3–36 months)	EL/RL REEL (36 months)	+
Mundy et al. (1995)	37	12–36 months	US: longitudinal	FM/GM BSID	EL RSLD (initial language)	+
				FM/GM BSID	RL RSLD (initial language)	+
				FM/GM BSID	EL RSLD (follow-up language)	+
				FM/GM BSID	RL RSLD (follow-up language)	+
Bird et al. (2008)	20	8–19 years	CA: longitudinal*	FM finger repetition	RL PPVT-R	None
Kanai et al. (2018)	78	4–6 years	JP: retrospective	FM/GM KSPD P-M DQ	EL/RL KSPD L-S DQ	+
Yamauchi et al. (2019)	156	10–43 months	JP: retrospective, longitudinal (subset)	FM/GM KSPD P-M DA	EL/RL KSPD L-S DA	+
				GM Walking Onset (1st to 2nd test)	EL/RL KSPD L-S DA (2nd test)	+
Locatelli et al. (2021)	105	3–17 years	IT: cross-sectional	GM Sitting Onset	EL Babbling Onset	+
				GM Walking Onset	EL Babbling Onset	+
				GM Sitting Onset	EL/RL VABS-II (Preschoolers)	−
				GM Sitting Onset	EL/RL WPPSI-III (School-Age)	+
Will and Roberts (2021)	37	10–44 months	US: cross-sectional	FM MSEL	EL VABS-II	+
				FM MSEL	RL VABS-II	+
				GM MSEL	EL VABS-II	+
				GM MSEL	RL VABS-II	+

*N*, number of children with Down syndrome in the study. CA, chronological age. Setting: CA, Canada. IT, Italy. JP, Japan. US, United States. Motor and Language Variables: BSID, Bayley scales of infant development (Motor). EL, expressive language. FM, fine motor. GM, gross motor. KSPD, Kyoto scale of psychological development where DA = developmental age, DQ = developmental quotient, L-S = language-social, and P-M = postural-motor. MSEL, Mullen scales of early learning (Gross Motor and Fine Motor). PPVT-R, Peabody picture vocabulary test-revised. REEL, receptive-expressive emergent language scale. RSLD, Reynell scales of language development. RL, receptive language. VABS-II, vineland adaptive behavior scales interview second edition (communication). WPPSI-III, Wechsler preschool and primary scale of intelligence Third edition (Verbal). Motor-language link: plus sign (+) denotes positive link(s), subtract sign (−) denotes negative link(s). * Only data from the second time point were reported in analyses.

Studies that used a global measure to index motor functioning unanimously found positive motor-language links in children with DS despite differences in study methods. In retrospective studies where children were assessed with the KSPD, motor and language development were positively correlated at 0–1 years, 2 years, 3 years, and 4–6 years (Kanai et al., 2018; Yamauchi et al., 2019). To address whether a specific motor gain may facilitate language, Yamauchi et al. (2019) examined a subset of their data that was tested twice with the KSPD and not walking at the first test. Walking achievement, controlling for initial language score and age at the second test, had a positive effect on children’s second language assessment. Will and Roberts (2021) further quantified motor-language links in a cross-sectional sample of children aged 10–44 months, reporting that every 1-point increase in motor skills captured by the MSEL was associated with a 1-point increase in language skills assessed with the VABS-II. This effect was independent of whether gross or fine motor scores were entered as the predictor, or if expressive or receptive language scores were the outcome variable, although motor and language functioning were sampled concurrently rather than longitudinally.

Two studies found positive correlations between early motor skills and later language skills. Reed et al. (1980) reported correlations that increased with age between motor functioning assessed with the BSID at 6, 12, 18, 24, 30, and 36 months and language functioning on the REEL at 36 months. In addition, muscle tone measured from 3–36 months as independent ratings of hypotonia from three specialists predicted language development at 36 months. Hypotonia may have unique implications for motor-language cascades in DS. Children without adequate muscle tone engage in fewer motor behaviors and consequently may have less opportunities to develop social skills, including language. In another study of children 12–36 months, Mundy et al. (1995) reported strong correlations between BSID motor age scores and initial expressive and receptive language on the RSLD, as well as follow-up language scores 13 months later. However, once initial age and initial language were controlled, motor age was not a significant source of variance for language outcomes. Mundy et al. (1995) measured hypotonia but did not test it as a separate language predictor. Given the limited evidence identified by this scoping review, more longitudinal research is needed before drawing conclusions regarding hypotonia and motor-language cascades in children with DS.

A final set of studies examined a specific motor skill with respect to language abilities, and these analyses yielded mixed results. A cross-sectional study by Locatelli et al. (2021) found a positive link between walking onset and babbling onset, supporting the longitudinal analyses by Yamauchi et al. (2019). A positive link between sitting onset and babbling onset was also found and sitting positively predicted language outcomes measured in school age children with the WPPSI-III. However, sitting was negatively related to language in a small subset of the sample who were preschoolers assessed with the VABS-II. Finally, Bird et al. (2008) found no link between finger repetition and receptive language on the PPVT-R, but this study was the most limited in scope relative to the objective of this scoping review as it did not address gross motor or expressive language skills.

## Discussion

This scoping review identified papers that have reported motor and language data in children with DS to map potential evidence for motor-language developmental cascades in this population. We considered that any longitudinal or concurrent links between motor and language skills in children with DS would build support for applying a developmental cascades lens to designing future studies. The findings of this review show that empirical evidence for motor-language links remains scant—most of the papers that reported both motor and language data did not analyze the link between domains. However, limited evidence where motor-language links were directly analyzed in longitudinal (Reed et al., 1980; Mundy et al., 1995; Yamauchi et al., 2019) and concurrent (Kanai et al., 2018; Yamauchi et al., 2019; Locatelli et al., 2021; Will and Roberts, 2021) study designs was largely positive.

Fewer papers were identified in this scoping review for children with DS relative to the similar review from Hwang and Lee (2022) on children with ASD, but trends were the same. Examining individual studies in more detail, motor skills in the first and second year of life predicted expressive language skills at 3 years of age in children with DS (Reed et al., 1980; Mundy et al., 1995). Leonard et al. (2015) found a similar pattern, reporting infants with ASD with more proficient motor skills at 7 months were more proficient in expressive language at 3 years. Two studies found a link between walking onset and later language in children with DS (Yamauchi et al., 2019; Locatelli et al., 2021). These findings agree with Bedford et al. (2016), who found that earlier walking onset in children with ASD was associated with greater expressive and receptive language development. Locatelli et al. (2021) also reported links between sitting onset and later language on two of three measures examined in children with DS. A prospective longitudinal study of children with an elevated likelihood for ASD and infants diagnosed with ASD found that a faster growth rate for pull-to-sit skills predicted speech scores at 24 months (Bradshaw et al., 2023). However, capturing change over time in a motor skill as a language predictor was missing from the DS literature.

Another study design that was not observed in the DS studies reviewed was the use of a motor milestone to predict change over time in language skills. In typically developing children, Oudgenoeg-Paz et al. (2012) reported that walking at a younger age predicted a higher rate of vocabulary growth from 16 to 28 months and Walle and Campos (2014) found that walking achievement, independent of age, was linked to a significant increase in expressive and receptive language. Together, evidence collected via different study designs in different developmental populations including children with DS supports motor-language cascades.

Why are motor skills important for later language skills? While the precise mechanisms are unknown, several possibilities exist for future hypothesis testing. Motor gains influence how the child interacts with objects and people in their environment. Delays in skills means less opportunities to explore objects and consequently less opportunities for learning (Iverson, 2021). Moreover, caregivers change how they interact with children based on the child’s motor skills (Biringen et al., 1995). Using sitting as an example, caregivers provided more encouragement to explore objects when children were sitting versus other postures (Kretch et al., 2022) and more object/action labels when children were exploring an object (Custode and Tamis-LeMonda, 2020; West et al., 2022). A delay in achieving a motor milestone such as sitting or a general deficit like hypotonia could mean not receiving these opportunities from caregivers, ultimately creating a consequence for language development. Additional studies guided by the developmental cascades framework are needed to test these possibilities for children with DS. While research in this area would benefit from longitudinal designs that correlate the emergence of a skill at one time with the emergence of a skill at another time point, only randomized controlled trials where motor experience is manipulated to see if an earlier motor skill onset leads to an earlier language skill onset can conclusively establish whether motor skills have a cascading influence on later language skills. The reviewed studies did not include this type of design.

## Limitations

One limitation of this scoping review is that only two developmental domains were examined. Development is dynamic, and motor functioning could have implications beyond language outcomes. For example, cognition is another area of need for children with DS. Motor-cognitive links were found in some of the papers reviewed (Kanai et al., 2018; Yamauchi et al., 2019). Infants with DS who spent more time exploring objects scored higher on cognitive skills (Fidler et al., 2019). Malak et al. (2013) found a strong connection between motor and cognitive development, particularly for the first 3 years; however, Kim et al. (2017) did not find a link between early motor skills and later cognitive functioning in children with DS. Moreover, Mundy et al. (1995) found links between motor skills and non-verbal communication skills such as social attention, joint attention, responding to joint attention, and nonverbal requests in children with DS. Possibilities for motor skills to interact with cognitive and social development should be considered in future research using the developmental cascades lens.

## Conclusion

Gains in motor skills are linked to gains in language skills in children with DS. This link has important clinical implications. As Lobo et al. (2013) suggest, if early motor interventions are to have long term effects, they should be done with the aim of promoting infants’ interactions with objects and people in larger family and societal contexts. Said another way, motor interventions cannot just be about motor functioning. We echo the call from Yamauchi et al. (2019) to examine the effect of motor interventions on language and cognitive outcomes. We encourage researchers to design prospective longitudinal studies to further characterize motor-language links in children with DS and to analyze motor and language links when such data are available in studies where motor-language cascades is not the primary goal. Finally, making motor and language data collected in children with DS publicly available for secondary analyses will further our understanding of motor-language cascades in this developmental population. Collectively, these efforts will inform the design of randomized controlled trials to test developmental cascades between motor and language skills in children with DS.

## Author contributions

AK: Conceptualization, Data curation, Funding acquisition, Methodology, Project administration, Writing – original draft, Writing – review & editing. EN: Conceptualization, Data curation, Methodology, Supervision, Writing – original draft, Writing – review & editing.

## Footnotes

^1^https://osf.io/dmxz7/; Karimi and Nelson (2023).
