# Supplementary material for: Motor-language links in children with Down syndrome: a scoping review to revisit the literature with a developmental cascades lens
Source: Front Psychol. 2023 Oct 2;14:1275325. doi: 10.3389/fpsyg.2023.1275325 (PMC10577202; doi:10.3389/fpsyg.2023.1275325)
Supplement: Supplementary file 1 [file Data_Sheet_1.docx]

**Supplementary Material**

Karimi & Nelson

*Motor-language links in children with Down syndrome: A scoping review to revisit the literature with a developmental cascades lens*

**Table S1.** Full search strings for each database.

| **Database** | **Key Search Terms** |
| --- | --- |
| PubMed | Search: ((Down syndrome) AND (Motor)) AND (language)  ("down syndrome"[MeSH Terms] OR ("down"[All Fields] AND "syndrome"[All Fields]) OR "down syndrome"[All Fields]) AND ("motor"[All Fields] OR "motor s"[All Fields] OR "motoric"[All Fields] OR "motorically"[All Fields] OR "motorics"[All Fields] OR "motoring"[All Fields] OR "motorisation"[All Fields] OR "motorised"[All Fields] OR "motorization"[All Fields] OR "motorized"[All Fields] OR "motors"[All Fields]) AND ("language"[MeSH Terms] OR "language"[All Fields] OR "languages"[All Fields] OR "language s"[All Fields] OR "programming languages"[MeSH Terms] OR ("programming"[All Fields] AND "languages"[All Fields]) OR "programming languages"[All Fields])  **Translations**  Down syndrome: "down syndrome"[MeSH Terms] OR ("down"[All Fields] AND "syndrome"[All Fields]) OR "down syndrome"[All Fields]  Motor: "motor"[All Fields] OR "motor's"[All Fields] OR "motoric"[All Fields] OR "motorically"[All Fields] OR "motorics"[All Fields] OR "motoring"[All Fields] OR "motorisation"[All Fields] OR "motorised"[All Fields] OR "motorization"[All Fields] OR "motorized"[All Fields] OR "motors"[All Fields]  language: "language"[MeSH Terms] OR "language"[All Fields] OR "languages"[All Fields] OR "language's"[All Fields] OR "programming languages"[MeSH Terms] OR ("programming"[All Fields] AND "languages"[All Fields]) OR "programming languages"[All Fields] |
| PsychInfo | (Down Syndrome) AND Motor AND language |
| OVID Medline | (Down syndrome and Motor and Language).mp. |

| **Table S2.** Studies that measured motor and language skills in children with DS but did not investigate motor-language links. | | | | | | | | |
| --- | --- | --- | --- | --- | --- | --- | --- | --- |
| **Source** | **N** | **CA** | **Setting: Design** | **GM** | **FM** | **EL** | **RL** | **Study Aims** |
| Neligan and Prudham (1969) | 20 | 1-3 yrs | UK: Longitudinal | Y | N | Y | N | To establish the predictive value of motor and language milestones for screening children at risk of intellectual disability |
| Melyn and White (1973) | 612 | 0-16 yrs | US: Longitudinal | Y | N | Y | N | To investigate the developmental milestones of children with DS reared at home versus institutions |
| Chen and Woolley (1978) | 96 | 2 mos - 8 yrs | US: Longitudinal | Y | Y | Y | Y | To construct a developmental assessment chart for non-institutionalized children with DS |
| Hogg and Moss (1983) | 50 | 15-44 mos | UK: Matched pairs | N | Y | Y | Y | To compare the development of fine motor skills in children with DS to typically developing children |
| Champion (1987) | 14 | 0-2 yrs | NZ: Longitudinal | Y | Y | Y | Y | To examine sensorimotor development in children with DS after an ecologically valid early intervention |
| Aparicló (1988) | 48 | 0-24 mos | ES: Experimental | Y | Y | Y | Y | To compare vicarious vs instructional training of parents providing early stimulation to children with DS |
| Van Dyke et al. (1990) | 21 | 2 mos - 19.2 yrs | US: Retrospective | Y | Y | Y | Y | To compare development across domains in individuals with DS who received cell therapy versus those who did not |
| Sanz and Menendez (1996) | 30 | 0-18 mos | ES: Longitudinal | Y | Y | Y | Y | To examine how the age of treatment onset influences later development in children with DS |
| Laws and Lawrence (2001) | 17 | 7.7-13.9 yrs | UK: Matched pairs | N | Y | N | Y | To compare spatial representation in the drawings of children with DS to typically developing children |
| Dolva et al. (2004) | 62 | 60-72 mos | NO: Cross-sectional | Y | Y | Y | Y | To describe functional performance and typical level of skill mastery in 5-year-old children with DS |
| Hernandez‐Reif et al. (2006) | 21 | 24.5 $\pm$ 9.5 mos | US: Experimental | Y | Y | Y | Y | To examine the effectiveness of a message therapy intervention on general development in children with DS |
| Visootsak et al. (2011) | 29 | 5.1-33.5 mos | US: Cross-sectional | Y | Y | Y | Y | To compare neurodevelopmental outcomes of children with DS with and without congenital heart disease |
| Wuang and Su (2011) | 206 | 6-13 yrs | TW: Cross-sectional | Y | Y | Y | Y | To investigate sensory processing profiles and visual organization abilities of children and adolescents with DS |
| Visootsak et al. (2013) | 29 | 25.9-40.5 mos | US: Cross-sectional | N | Y | Y | Y | To examine whether congenital heart disease influence language skills in children with DS |
| Silva et al. (2013) | 20 | 5-45 mos | US: RCT | Y | Y | Y | Y | To examine whether parent-delivered massage improves expressive language in children with DS |
| Fuengfoo and Sakulnoom (2014) | 210 | 0-3 yrs | TH: Prospective | Y | Y | Y | Y | To examine the influence of early intervention on developmental outcomes in children with DS |
| Ferreira-Vasques and Lamonica (2015) | 20 | 2 mos - 6 yrs | BR: Matched pairs | Y | Y | Y | Y | To compare motor, social and language skills in children with DS with matched typically developing children |
| Roncadin et al. (2015) | 26 | 4-17 yrs | CA: Cross-sectional | N | Y | Y | Y | To compare the neuropsychological performance of children with DS with and without a history of leukemia |
| Tapp et al. (2015) | 29 | 28.5 ± 6.3 mos | US: Cross-sectional | Y | Y | Y | Y | To compare the neurodevelopmental outcomes of children with DS with and without infantile spasm |
| Alsaied et al. (2016) | 178 | 0-18 yrs | US: Retrospective cohort | Y | Y | Y | Y | To examine the effect of congenital heart disease and cardiac surgery in the first year of life on developmental outcomes of children with DS |
| Visootsak et al. (2016) | 57 | 12-14 mos | US: Cross-sectional | Y | Y | Y | Y | To examine the mediating role of family and maternal factors on the neuropsychological outcomes of children with DS with congenital heart disease |
|  |  |  |  |  |  |  |  |  |
| *Table S1 continued* |  |  |  |  |  |  |  |  |
| **Source** | **N** | **CA** | **Setting: Design** | **GM** | **FM** | **EL** | **RL** | **Study Aims** |
| Aoki et al. (2018) | 158 | 10-48 mos | JP: Longitudinal | Y | Y | Y | Y | To examine the influence of preterm birth, low birth weight and congenital heart disease on motor, cognitive and language of children with DS |
| Arango et al. (2018) | 31 | 15-80 mos | CL: Cross-sectional | Y | Y | Y | Y | To compare the developmental trajectories of children with DS with matched typically developing children |
| D'Souza et al. (2020) | 38 | 8-48 mos | US: Cross-sectional | Y | Y | Y | Y | To examine the relation between prenatal depression and motor and language development in children with DS with a focus on expressive language |
| Kokkoni et al. (2020) | 1 | 11 mos | US: Case study | Y | Y | Y | Y | To examine the influence of a portable body weight support system on the development of a child with DS |
| Pinero-Pinto et al. (2020) | 32 | 4-8 mos | ES: RCT | Y | Y | Y | Y | To determine the effects of massage therapy on the global development of children with DS |
| *Note.* If the age range was not given in the paper, the sample mean and standard deviation were reported.  DS, Down syndrome. N, Number of children with Down syndrome in the study. CA, Chronological Age. Mos., months. Yrs., years. RCT = Randomized Controlled Trial. Setting: BR, Brazil. CA, Canada. CL, Chile. ES, Spain. JP, Japan. NZ, New Zealand. NO, Norway. TW, Taiwan. TH, Thailand. UK, United Kingdom. US, Unites States. Motor and Language Outcomes: GM, Gross motor. FM, Fine motor. EL, Expressive language. RL, Receptive language. Y, yes reported. N, no. | | | | | | | | |

**References:**

Alsaied, T., Marino, B. S., Esbensen, A. J., Anixt, J. S., Epstein, J. N., & Cnota, J. F. (2016). Does congenital heart disease affect neurodevelopmental outcomes in children with Down syndrome? *Congenital heart disease*, *11*(1), 26-33. <https://doi.org/10.1111/chd.12322>

Aoki, S., Yamauchi, Y., & Hashimoto, K. (2018). Developmental trend of children with Down’s syndrome–How do sex and neonatal conditions influence their developmental patterns? *Brain and Development*, *40*(3), 181-187. <https://doi.org/10.1016/j.braindev.2017.10.001>

Aparicló, M. T. S. (1988). Effect of observational training of parents in the early stimulation of trisomy‐21 babies. *Early Child Development and Care*, *41*(1), 89-101. <https://doi.org/10.1080/0300443880410108>

Arango, P. S., Aparicio, A., & Tenorio, M. (2018). Developmental trajectories of children with Down syndrome by socio-economic status: the case of Latin America. *J Intellect Disabil Res*, *62*(9), 759-774. <https://doi.org/10.1111/jir.12516>

Champion, P. (1987). An investigation of the sensorimotor development of Down's Syndome infants involved in an ecologically based early intervention programme: a longitudianl study *The British Journal of Mental Subnormality*, *33*(65), 88-99. <https://doi.org/10.1179/bjms.1987.012>

Chen, H., & Woolley, P. V., Jr. (1978). A developmental assessment chart for non-institutionalized Down syndrome children. *Growth*, *42*(2), 157-165.

D'Souza, H., Lathan, A., Karmiloff-Smith, A., & Mareschal, D. (2020). Down syndrome and parental depression: A double hit on early expressive language development. *Res Dev Disabil*, *100*, 103613. <https://doi.org/10.1016/j.ridd.2020.103613>

Dolva, A. S., Coster, W., & Lilja, M. (2004). Functional performance in children with Down syndrome. *Am J Occup Ther*, *58*(6), 621-629. <https://doi.org/10.5014/ajot.58.6.621>

Ferreira-Vasques, A. T., & Lamonica, D. A. (2015). Motor, linguistic, personal and social aspects of children with Down syndrome. *J Appl Oral Sci*, *23*(4), 424-430. <https://doi.org/10.1590/1678-775720150102>

Fuengfoo, A., & Sakulnoom, K. (2014). Clinical abnormalities, early intervention program of Down syndrome children: Queen Sirikit National Institute of Child Health experience. *J Med Assoc Thai*, *97 Suppl 6*, S152-158. <https://www.ncbi.nlm.nih.gov/pubmed/25391188>

Hernandez‐Reif, M., Field, T., Largie, S., Mora, D., Bornstein, J., & Waldman, R. (2006). Children with Down syndrome improved in motor functioning and muscle tone following massage therapy. *Early Child Development and Care*, *176*(3-4), 395-410. <https://doi.org/10.1080/03004430500105233>

Hogg, J., & Moss, S. (1983). Prehensile development in Down's syndrome and non‐handicapped preschool children. *British Journal of Developmental Psychology*, *1*(2), 189-204. <https://doi.org/10.1111/j.2044-835X.1983.tb00557.x>

Kokkoni, E., Stoner, T., & Galloway, J. C. (2020). In-Home Mobility Training With a Portable Body Weight Support System of an Infant With Down Syndrome. *Pediatr Phys Ther*, *32*(4), E76-E82. <https://doi.org/10.1097/PEP.0000000000000752>

Laws, G., & Lawrence, L. (2001). Spatial representation in the drawings of children with Down's syndrome and its relationship to language and motor development: A preliminary investigation. *British Journal of Developmental Psychology*, *19*(3), 453-473. <https://doi.org/10.1348/026151001166119>

Melyn, M. A., & White, D. T. (1973). Mental and developmental milestones of noninstitutionalized Down's syndrome children. *Pediatrics*, *52*(4), 542-545. <https://doi.org/10.1542/peds.52.4.542>

Neligan, G., & Prudham, D. (1969). Potential value of four early developmental milestones in screening children for increased risk of later retardation. *Dev Med Child Neurol*, *11*(4), 423-431. <https://doi.org/10.1111/j.1469-8749.1969.tb01460.x>

Pinero-Pinto, E., Benitez-Lugo, M. L., Chillon-Martinez, R., Rebollo-Salas, M., Bellido-Fernandez, L. M., & Jimenez-Rejano, J. J. (2020). Effects of Massage Therapy on the Development of Babies Born with Down Syndrome. *Evid Based Complement Alternat Med*, *2020*, 4912625. <https://doi.org/10.1155/2020/4912625>

Roncadin, C., Hitzler, J., Downie, A., Montour-Proulx, I., Alyman, C., Cairney, E., & Spiegler, B. J. (2015). Neuropsychological late effects of treatment for acute leukemia in children with Down syndrome. *Pediatr Blood Cancer*, *62*(5), 854-858. <https://doi.org/10.1002/pbc.25362>

Sanz, M., & Menendez, J. (1996). A study of the effect of age of onset of treatment on the observed development of Down's syndrome babies. *Early Child Development and Care*, *118*(1), 93-101. <https://doi.org/10.1080/0300443961180108>

Silva, L. M. T., Schalock, M., & Williams, M. (2013). Improved speech following parent-delivered qigong massage in young children with Down syndrome: a pilot randomised controlled trial. *Early Child Development and Care*, *183*(12), 1891-1905. <https://doi.org/10.1080/03004430.2012.761607>

Tapp, S., Anderson, T., & Visootsak, J. (2015). Neurodevelopmental outcomes in children with Down syndrome and infantile spasms. *J Pediatr Neurol*, *13*(2), 74-77. <https://doi.org/10.1055/s-0035-1556768>

Van Dyke, D. C., Lang, D. J., van Duyne, S., Heide, F., & Chang, H. J. (1990). Cell therapy in children with Down syndrome: a retrospective study. *Pediatrics*, *85*(1), 79-84. <https://doi.org/10.1542/peds.85.1.79>

Visootsak, J., Hess, B., Bakeman, R., & Adamson, L. B. (2013). Effect of congenital heart defects on language development in toddlers with Down syndrome. *J Intellect Disabil Res*, *57*(9), 887-892. <https://doi.org/10.1111/j.1365-2788.2012.01619.x>

Visootsak, J., Huddleston, L., Buterbaugh, A., Perkins, A., Sherman, S., & Hunter, J. (2016). Influence of CHDs on psycho-social and neurodevelopmental outcomes in children with Down syndrome. *Cardiol Young*, *26*(2), 250-256. <https://doi.org/10.1017/S1047951115000062>

Visootsak, J., Mahle, W. T., Kirshbom, P. M., Huddleston, L., Caron-Besch, M., Ransom, A., & Sherman, S. L. (2011). Neurodevelopmental outcomes in children with Down syndrome and congenital heart defects. *Am J Med Genet A*, *155A*(11), 2688-2691. <https://doi.org/10.1002/ajmg.a.34252>

Wuang, Y. P., & Su, C. Y. (2011). Correlations of sensory processing and visual organization ability with participation in school-aged children with Down syndrome. *Res Dev Disabil*, *32*(6), 2398-2407. <https://doi.org/10.1016/j.ridd.2011.07.020>
